# Relationship between electrolytes and glycated hemoglobin among diabetic patients with poor adherence to antidiabetic medications: a cross-sectional study

**DOI:** 10.11604/pamj.2024.47.37.41815

**Published:** 2024-01-30

**Authors:** Francis Lengeiya, Scholastica Mathenge, Patroba Ojola

**Affiliations:** 1 Department of Medical Laboratory Sciences, Kenyatta University, Nairobi, Kenya,; 2 Department of Microbiology and Biotechnology, Kenyatta University, Nairobi, Kenya,

**Keywords:** Glycated hemoglobin, type 2 diabetes mellitus, electrolytes, antidiabetics, adherence, management, healthcare, correlation

## Abstract

**Introduction:**

type 2 Diabetes mellitus is a chronic metabolic disease with devastating effects on patients and results in numerous healthcare challenges in terms of its management and the cost burden among the affected. Successful management involves maintaining optimal glycemic control to prevent complications, with adherence to antidiabetic medications playing a crucial role in achieving this objective. Additionally, maintaining a healthy electrolyte balance is key for overall well-being and physiological function. However, the correlation between glycated hemoglobin and electrolyte balance remains under investigated, particularly in patients with suboptimal adherence. The aim of this research was to study the relationship between glycated hemoglobin and electrolytes among diabetic patients with poor adherence to antidiabetic medications.

**Methods:**

this study was conducted at Samburu County Referral Hospital in Samburu County, Kenya. We employed a descriptive cross-sectional design focusing on adult diabetic patients aged 18 years and above who had visited the diabetic clinic over a three-month period. To evaluate their adherence levels, we employed a Morisky Medication Adherence Scale-8. Seventy-two diabetic patients who got adherence level scores of < 6 were categorized as having low adherence and their blood samples were collected for measuring glycated hemoglobin levels and electrolytes levels particularly potassium, sodium, calcium, magnesium, phosphorus and chloride. Relationship between electrolytes and glycated hemoglobin among diabetic patients with poor adherence to antidiabetics was determined using Karl Pearson correlation.

**Results:**

among the study participants, the lowest hemoglobin A1C (HbA1c) level recorded was 5.1% while the highest was 15.0% and the majority (41.7%) fell within the HbA1c range of 5-7%. A high proportion of individuals (58.3%) with poor adherence to antidiabetics had elevated HbA1c levels, indicating poor glycemic control. The correlations observed between glycated hemoglobin and electrolytes which included magnesium, sodium, chloride, calcium and phosphorus was r= -0.07, -0.32, -0.05 -0.24 and -0.04 respectively.

**Conclusion:**

this study concluded that there is a relationship between electrolytes and glycated hemoglobin among diabetic patients with poor adherence to antidiabetics. A statistically significant negative correlation was observed between glycated hemoglobin and calcium level (r=-0.2398 P ≤0.05) and also sodium (r=-0.31369 P≤0.05). A negative correlation (P≥0.05) was observed between phosphorus, magnesium, chloride and potassium with HbA1c levels though not statistically significant.

## Introduction

Type 2 diabetes mellitus is a chronic metabolic disorder which results in numerous health care challenges in terms of its management and the cost burden among the affected. Central challenge to effective management of type 2 diabetes mellitus is the imperative to achieve and sustain optimal glycemic control, a cornerstone in preventing the debilitating complications associated with the condition. Elevated levels of HbA1c among diabetic patients, have been associated with peripheral neuropathy, cardiac diseases, retinal vascular disease and renal diseases [1]. Glycated hemoglobin (HbA1c) levels can be used to determine and predict level of blood sugar. It estimates average blood glucose for the past 3 months and can also be used as a diagnostic test [2]. However, the intricate interplay between glycated hemoglobin levels and electrolyte imbalances in patients with poor adherence to antidiabetic medications remains an area of limited exploration.

It is well recognized that poor adherence to prescribed antidiabetics may lead to poor glycemic control, which could result in micro-vascular and macro-vascular complications [3]. Inadequate commitment to and compliance with antidiabetic medication places patients at risk of complications, potentially leading to failure in achieving glycemic control objectives. The issue hinders optimal diabetes mellitus management and remains a significant challenge to address [4]. The ramifications of such nonadherence extend beyond mere glycemic fluctuations, potentially influencing a broader range of metabolic processes, including electrolyte homeostasis. The levels of electrolytes are mostly determined to assess patients` clinical conditions and any imbalance results in electrolyte disorders. Dysregulation of electrolyte distribution caused by hyperglycemic osmotic fluid shift results in electrolyte imbalance [5] limiting T2DM treatment. It is therefore, necessary to explore the relationship between electrolytes and extended blood sugar level status through testing of glycated hemoglobin levels in diabetic patients who do not adhere to the antidiabetic treatment regimen.

Through a cross-sectional study, we sought to unravel the relationship between HbA1c levels and electrolyte levels (sodium, potassium, chloride, magnesium, calcium and phosphorus) in individuals struggling with medication adherence. By delving to this unexplored territory, we aimed to unearth valuable insights that hold the potential to inform tailored interventions and enhance the overall care of patients grappling with diabetes. This study´s findings stand to bridge the gap in knowledge, fostering a deeper understanding of how glycemic control and electrolyte balance intertwine in the context of medication adherence challenges. Ultimately, our pursuit is not only to expand the scientific discourse but also to contribute to the optimization of diabetes management strategies for those in need.

**General objective:** to determine the relationship between electrolytes and glycated hemoglobin among diabetic patients with poor adherence to antidiabetics attending Samburu County Referral Hospital, Kenya.

**Specific objectives:** i) to determine glycated hemoglobin (HbA1C) levels among diabetic patients with poor adherence to antidiabetics; ii) to determine the levels of chloride, potassium, calcium, magnesium, phosphate and sodium electrolytes and their correlation with glycated hemoglobin levels among diabetic patients with poor adherence to antidiabetics.

## Methods

**Study area:** the study was carried out at Samburu County Referral Hospital in Samburu County with GPS coordinates 1.0981° N, 36.6962° E. The hospital is a level 5 hospital serving residents across Samburu County and remains the largest hospital in the county with a 200 inpatient bed capacity and a specialized diabetes clinic.

**Study design:** the study used a descriptive cross-sectional study design to adult diabetic patients who attended Samburu County Referral Hospital Outpatient diabetes clinic.

**Study population:** the study involved adult diabetic patients who attended diabetic outpatient clinic at Samburu County Referral Hospital.

**Inclusion criteria:** diabetic patients aged above 18 years under antidiabetics for at least the preceding 3 months and should have low adherence to the medications (score≤6) on Morisky Medication Adherence Scale.

**Exclusion criteria:** this study excluded diabetic patients below 18 years of age who attended the outpatient diabetic clinic, as well as those who had not been under antidiabetic treatment for at least 3 months and diabetic patients who were unwilling to give consent and participate in the study. Additionally, individuals with a Morisky Medication Adherence scale score of ≥6 were also excluded.

**Sampling procedure and Sample size determination:** to ensure a representative sample, we employed a systematic sampling method, selecting every 3^rd^ patient on a predefined schedule. Eligible individuals to participate were selected based on the inclusion criteria mentioned in section 2.3.1 We explained to them the study´s purpose, and sought their consent for participation. The sample size determination was done using formula [6]:

$$n=\frac{Z^{3}pq}{d^{2}}$$

Where; n= the desired sample size; Z= 95% confidence interval or 1.96; d= degree of accuracy set at 0.05 levels; P= proportion in target population estimated to have characteristics being measured where P= 0.05 (a recent study done in Kenya reports T2DM prevalence to be at 5% [7]; q= is the complement of (1.0-p). Using this formular, the calculated sample size (n) was found to be 72 participants.

**Morisky medication adherence scale-8 data collection instrument:** recruitment also involved administering the Morisky Medication Adherence Scale-8 questionnaire to assess adherence levels [8].

**Validity and reliability of MMAS-8:** Morisky Medication Adherence Scale (MMAS-8) is an assessment tool for adherence to medications across patient populations [8]. The tool uses a number of behavioral questions that are structured to prevent yes-saying responses that may result in biased outcomes. Morisky Medication Adherence Scale-8 contains 8 questions, the responses for each item are yes/no and every item has a dichotomous response (0 and 1 scores). Scores range from 0 to 8. Adherence was then gauged; a score of 8 was high adherence, a score of 7 was regarded as medium adherence while a score of below 6 was low adherence. For those patients who did not understand English language, the questions were interpreted into languages they best understood for instance, Samburu or Swahili languages.

**Laboratory analysis of HbA1c:** the Standard Operating Procedures (SOPs) were adhered to, in pre-analytical phase by collecting sufficient amount of sample, at least 3mls of blood for measuring serum electrolytes and at least 3mls for measuring glycosylated hemoglobin levels. Request forms were filled properly with unique patient identification numbers. Blood samples for measuring HbA1c levels were collected in sodium fluoride vacutainers and transported to the laboratory for analysis using automated HbA1c analyzer machine which uses a high-performance liquid chromatographic (HPLC) technique to determine HbA1c concentration.

**Specimen analysis:** the “standard test” mode was selected on the analyzer and operator ID was input after which the test device was inserted into the slot. Five microliter of blood was collected with the foil automatically through capillary action. The edge of foil was inserted into the extraction buffer and mixed well by pressing and releasing rubber for 6-8 times carefully and slowly to avoid bubble from forming. All the specimen was collected and applied on the well of the test device already inserted into the analyzer. And pressed “TEST START” button. The analyzer displayed results immediately after 3 minutes.

**Laboratory analysis of electrolytes:** venous blood for determination of serum electrolytes was collected in plain red-top vacutainers, labeled properly and delivered into the laboratory immediately. Upon delivery to the laboratory the samples were allowed to settle to allow complete clotting then put in a centrifuge and spun at 3000rpm for 5 minutes, the serum was transferred into sample cups and labeled appropriately. The samples were analyzed for electrolyte levels (sodium, calcium, phosphorus, potassium and chlorides). This was done by use of automated Selectra prom biochemistry analyzer which is based on Ion Selective Electrode (ISE) method. The results were then recorded.

**Quality assurance:** Samburu County Referral Hospital Biochemistry Laboratory runs daily internal and external quality control on all tests. To ensure quality of analysis two levels of Internal Quality Control (IQC) were conducted by first calibrating the Selectra prom biochemistry analyzer and doing control check using a control sample which had to meet the acceptable values before the samples were analyzed. Calibration set tests were performed to ensure standard F HbA1c analyzer produced accurate results. External quality control tests were run to ensure performance of analyzer and test device and production of quality results.

**Data analysis:** the completed questionnaires on medication adherence were numbered and coded for the case of handling. The results were presented in form of bar graphs, scatter plots and frequency tables. Relationship between impaired electrolytes and glycated hemoglobin due to poor adherence to antidiabetics were determined using Karl Pearson correlation.

**Ethical considerations:** ethical clearance and permission to conduct this study was sort from the KU Ethical Review Committee, NACOSTI and from SCRH Administration. Confidentiality was observed by not sharing information with any unauthorized person and the patient identity was protected by using assigned unique numbers instead of name.

## Results

**Description of the study subjects:** seventy-two study participants (48.6% females, 51.4% males) were enrolled in the study. The age range was 21 to 88 years, with a mean of 53.9 and a median of 53.5 years. The majority were married (72.7%), rural residents (80%), and had at least a secondary school education (30.3%) (Table 1).

**Table 1:** demographic data of the participants

Variable	Features	Frequency	Percentage
**Gender**	Male	37	51.4%
	Female	35	48.6%
**Age (years)**	18-29	2	2.8%
	30-49	24	33.3%
	50-64	26	36.1%
	65and above	20	27.8%
**Marital status**	Married	60	83.3%
	Single	12	16.7%
**Education level**	No education	10	13.9%
	Primary level	17	23.6%
	Secondary level	27	37.5%
	Tertiary level	18	25.0%
**Residence**	Urban	30	41.7%
	Rural	42	58.3%

**Figure 1:**
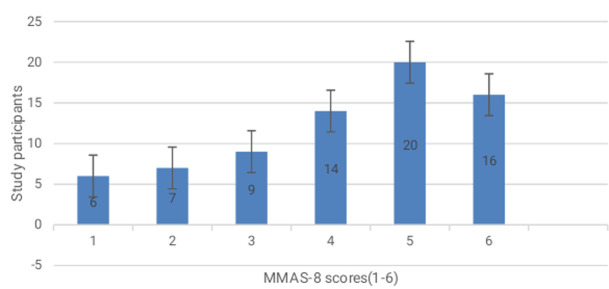
a bar graph showing study participants against MMAS-8 scores

**MMAS-8 questionnaire scores:** MMAS-8 scores ranged from 1-6. The majority (20 participants) scored 5, while the fewest (6 participants) scored 1. Other scores included 2 (7 participants), 3 (9 participants), and 6 (16 participants) (Figure 1).

**Glycated hemoglobin levels:** the HbA1c levels ranged from 5.1% to 15.0%. Notably, 41.7% fell within the 5-7% range, indicating acceptable glycemic control despite poor adherence. Additionally, 22.2% had levels in the 8-10% range, while 23.6% and 12.5% fell within 11-13% and 14-16% ranges, respectively. The mean HbA1c level was 9.38% with a standard deviation of 3.16 and a median of 8.96% (Table 2).

**Table 2:** frequency distribution table on HbA1c levels among diabetic patients with poor adherence to antidiabetics

Hemoglobin A1C (HbA1c) levels (%)	Frequency	Relative frequency	Cumulative frequency
5-7	30	41.7%	30
8-10	16	22.2%	46
11-13	17	23.6%	63
14-16	9	12.5%	72

**Correlation between HbA1c and electrolytes levels:** to explore the relationship between HbA1c and electrolyte levels, we conducted Pearson correlation analyses (Table 3). A moderate negative correlation of -0.32 with sodium and calcium -0.24 was observed, demonstrating statistical significance (P<0.05). However, correlations with potassium, chloride, magnesium and phosphorus were weak and lacked statistical significance (all p>0.05). This focused analysis enhances our understanding of the interplay between HbA1c and specific electrolytes.

**Table 3:** correlation between HbA1c and electrolytes levels

		Na+	K+	Cl-	Ca	Mg	Phos
Hemoglobin A1C (HbA1c) levels	Pearson correlation	-0.32	-0.01	0.05	-0.24	-0.07	-0.04
	R square	0.102	0.000	0.002	0.058	0.005	0.002
	Sig.	<0.05	>0.05	>0.05	<0.05	>0.05	>0.05
	N	72	72	72	72	72	72

Na+: sodium; K+: potassium; Cl-: chlorine; Ca: calcium; Mg: magnesium; Phos: phosphorus; Sig. = significance N= number

## Discussion

**Demographic data of the study participants:** in this study, the highest number of participants were in the age bracket of 50-64 years, making up 36.1% of the total. The lowest number of participants, at 2.8% belonged to the 18-29 years age group. Those aged 65 years and above accounted for 27.8% of the study participants, as shown in Table 1. Older participants in the age groups of 50-64 years and 65 and above are more likely to have had diabetes for a longer duration. This extended duration of the condition can introduce challenges related to comorbidities, polypharmacy (the use of multiple medications), and age-related cognitive decline, all of which may impact their ability to prescribe medications. The table also shows that the participants are almost evenly distributed by gender, with 51.4% males and 48.6% females. Studies have shown that men and women may have different approaches when it comes to managing chronic conditions. For instance, females might be more diligent in taking medications regularly, while males may be less adherent.

**Glycated hemoglobin (HbA1C) levels among diabetic patients with poor adherence to antidiabetics:** the results indicate that a substantial proportion of the study participants demonstrated suboptimal glycemic control, as evidenced by elevated HbA1c levels (%). Among the participants, only 23% had HbA1c levels within the target range of 5-7% (Table 2). This finding is comparable to the results by Kibirige *et al*. [9] where suboptimal glycemic control was noted in 311 study participants, accounting for 73.52% of the study participants [10] reported a slightly lower percentage (54.8%) of suboptimal glycemic control among diabetic patients and this indicates that a significant majority of patients who were poorly adhering to their antidiabetic medications had difficulty achieving and maintaining optimal glycemic control.

The finding that 14% of the participants had HbA1c levels ranging from 7-9% (Table 2) suggests a moderate level of hyperglycemia among this population. The percentage of study participants within that range was reported to be slightly lower (13.24%) by Kibirige *et al*. [9]. It implies that many individuals who were non-compliant with their antidiabetic medications experienced challenges in controlling their blood glucose levels. This finding emphasizes the detrimental impact of poor adherence on glycemic control and highlights the need for interventions to improve medication adherence and enhance overall treatment outcomes. Furthermore, 9% of the participants had HbA1c levels between 9-11% (Table 2), indicating poor glycemic control and a higher risk of developing complications associated with diabetes [9] reported a higher percentage (17.97%) of study participants within the range. This finding underscores the significance of addressing medication adherence issues among this subgroup. Effective interventions aimed at enhancing patient education, support and motivation to adhere to their prescribed antidiabetic medications are crucial to achieving better glycemic control and reducing the risk of long-term complications.

The data also reveals that 15% of the study participants had HbA1c levels ranging from 11-13% (Table 2) indicating significantly elevated blood glucose levels. In addition, 10% and 1% of the participants had HbA1c levels between 13-15%, and 15-17% respectively as shown on the same table, signifying extremely poor glycemic control. This finding highlights the critical nature of the problem of poor adherence among this population. Intensive interventions, including close monitoring, education, and involving family members or caregivers, are necessary to support patients in achieving better medication adherence and improving their glycemic control.

**Levels of electrolytes (chloride, potassium, calcium, magnesium, phosphorus and sodium) and correlation with HbA1c among diabetic patients with poor adherence to antidiabetics:** electrolyte imbalance is usually common among patients who adhere poorly to antidiabetics. This depends on many factors but most common causes are hyperglycemia and diabetic ketoacidosis [11]. This study showed a statistically significant (P≤0.05) negative correlation of -0.31369 between sodium level and glycated hemoglobin (Table 3). It can therefore be stated that an increase in glycated hemoglobin has a consequent decreasing effect on the level of sodium among type 2 diabetes patients. Similarly, Khan *et al*. [12] observed a decrease in serum sodium in uncontrolled diabetes mellitus and observed sodium levels to be highly significant (P-≤0.05). These findings can be explained by several underlying mechanisms. Firstly, the kidneys respond to high blood glucose by initiating osmotic diuresis, increasing urine production to eliminate excess glucose. As a consequence, water and electrolytes, including sodium ions, are excreted in higher amounts, leading to a reduction in serum sodium levels. Moreover, the increased urine production can result in dehydration if fluid intake does not match the elevated fluid loss. Dehydration also causes a concentration of sodium in the blood, further contributing to lower serum sodium levels.

In this study, it was found that there was a negative correlation between HbA1c and chloride levels, r=-0.04815 (Table 3). However, the correlation is not statistically significant (P≥0.05) indicating that changes in chloride levels are unlikely to have a notable impact on HbA1c levels. These findings are comparable with results by Santhosh *et al*. [13] which showed insignificant alterations of chloride levels in type 2 diabetic individuals. A contrasting finding was seen in a study conducted by Ogunleye *et al*. [14] whereby his study had shown that type 2 diabetic patients had lower levels of chloride, potassium, magnesium and sodium and phosphorus and he explained that the loss of serum electrolytes occurred due to increased excretion of those electrolytes in urine or as a result of lowered absorption. This study comprised of mostly stable individuals with relatively well-controlled diabetes and this might have minimized potential interference of chloride levels.

A negative correlation between potassium electrolyte and glycated hemoglobin was observed, r=-0.01188 (P≥0.05) as shown in Table 3. It can therefore be stated that increase in glycated hemoglobin is inversely related to the potassium level in the body. The lack of statistical significance suggests that changes in potassium levels are not strongly associated with changes in HbA1c levels and since our study participants were on diabetes treatment regimens, this could contribute to heterogeneity and obscure potential alterations in potassium levels. This observation is in agreement with a study by Yeemard *et al*. [10] which also found no correlation between potassium level and glycated hemoglobin. Although significant variations have been shown in a study conducted by Liamis *et al*. [11] whereby increased serum potassium levels were found to be statistically highly significant (P≤0.05) and he explained that the movement of potassium from the inside of cells to the outside, can result in hyperkalemia which is due to renal dysfunction, insufficient insulin levels, or hypertonicity.

A statistically significant (P≤0.05) negative correlation coefficient of -0.2398 between HbA1c concentration and calcium level was observed in this study (Table 3). This suggests that higher calcium levels may be associated with lower HbA1c levels. Insulin resistance, a hallmark of type 2 diabetes, can disrupt calcium metabolism. Normally, insulin promotes calcium uptake by cells, including bone cells. However, in insulin-resistant states, the effectiveness of insulin is reduced, leading to impaired calcium transport into cells. As a result, individuals with type 2 diabetes may experience lower calcium levels. Additionally, chronic kidney disease (CKD) is a common complication of type 2 diabetes and can further contribute to decreased calcium levels. Chronic kidney disease (CKD) affects the kidney´s ability to regulate calcium levels by impacting filtration, reabsorption, and excretion of calcium. Consequently, kidney dysfunction in individuals with type 2 diabetes can disrupt calcium homeostasis and lead to lower calcium levels. These findings are comparable with findings of a study done by Eshetu *et al*. [15] where it was observed that mean serum calcium level for type 2 diabetes mellitus individuals reduced when compared to that of the controls and showed a significant negative correlation between serum calcium levels and glycated hemoglobin.

This study showed a negative correlation, r=-0.06897, between magnesium level and HbA1c concentration although the association was not statistically significant (P≥0.05) (Table 3). Therefore, suggesting that changes in magnesium levels may not strongly influence HbA1c levels. The non-significant correlation between magnesium level and HbA1c concentration observed in this study may be because most of our participants are stable patients who visit the outpatient clinic hence limited cases of renal failure. This study is in contrast with some studies which found a significant negative correlation between glycated hemoglobin and magnesium level. Wang *et al*. [16] observed a decrease in serum magnesium levels and a significant negative correlation with HbA1c (Std β=-0.34 P <0.01) in Chinese subjects with diabetes. The study further explained that in insulin-resistant states, the impaired insulin function may disrupt magnesium transport into cells, leading to lower magnesium levels in the bloodstream. It therefore, concluded that diabetic patients who experienced macrovascular complications had significantly lower serum magnesium levels compared to those without macrovascular complications.

The serum level of phosphorus was found to have a negative correlation of -0.04016 with HbA1c levels but the alteration was not statistically significant (P>0.05) (Table 3). This implies that alterations in phosphorus levels are unlikely to have a substantial impact on HbA1c levels. A study by Fang *et al*. [17] similarly found no significant correlation between the level of blood glucose and serum level of phosphorus in type 2 diabetic group however, he observed that the blood glucose and serum levels of phosphorus were positively correlated (r= 0.226, P=0.042) and concluded that there was noticeable decrease in serum phosphorus levels, suggesting a potential disturbance in phosphorus metabolism. The treatment of diabetic patients often involves medications and interventions targeting blood glucose management. These treatments can have an impact on phosphorus metabolism, as certain antidiabetic medications or adjunct therapies can directly or indirectly affect phosphorus levels. The complexity of treatment approaches and medication regimens introduces additional variability, which may mask the correlation between serum phosphorus and HbA1c levels as observed in our study.

**Study limitations:** some patients had more than one comorbidity which may also affect the association between serum electrolytes and glycated hemoglobin. It is a cross-sectional study and the sample size is small. Large scale clinical trials are needed in future to determine the specific mechanism through which the relationship between serum electrolytes and glycated hemoglobin exists. Seven study participants (9.7%) who scored poorly on MMAS-8 surprisingly had good glycemic control <6% HbA1c. This may have had some effect on result accuracy.

## Conclusion

This study concludes that a high proportion of individuals with poor adherence to antidiabetics had elevated HbA1c levels, indicating poor glycemic control. The study also found out that there is statistically significant correlations (P≤0.05) observed between glycated hemoglobin levels with sodium (r=-0.31369) and calcium (r=-0.2398) levels in diabetic patients with poor glycemic control. This study provides insight into the complex interplay between glycated hemoglobin levels, medication adherence, and electrolyte imbalances in diabetic patients. The results highlight the importance of comprehensive diabetes management strategies that address both glycemic control and electrolyte status.

### What is known about this topic

- *Glycated hemoglobin as a long-term indicator of blood sugar control in diabetes;*
- *Fluctuations in electrolytes can affect insulin sensitivity and glucose control*.

### What this study adds

- *Enhanced specificity on correlation: this study refines its contribution by elucidating a significant negative correlation (P≤0.05) between HbA1c levels and the concentration of sodium and calcium in diabetic patients; emphasizing not just statistical significance but the depth and nature of this correlation provides a more nuanced understanding of the intricate relationships within diabetes physiology;*
- *Emphasis on clinical implications: moving beyond statistical metrics, our findings stress the clinical relevance of the identified correlation; the intricate relationship between HbA1c, sodium, and calcium levels calls for a reevaluation of diabetes management strategies;*
- *This study serves as a catalyst for a paradigm shift towards comprehensive care, highlighting the need to address both glycemic control and electrolyte status for optimized patient outcomes*.
